# Integration of Metabolomics and Transcriptomics Reveals Major Metabolic Pathways and Potential Biomarkers Involved in Pulmonary Tuberculosis and Pulmonary Tuberculosis-Complicated Diabetes

**DOI:** 10.1128/spectrum.00577-23

**Published:** 2023-07-31

**Authors:** Yunguang Wang, Xinxin He, Danna Zheng, Qiang He, Lifang Sun, Juan Jin

**Affiliations:** a Department of Critical Care Medicine, Affiliated Hangzhou First People’s Hospital, Zhejiang University School of Medicine, Hangzhou, Zhejiang, People’s Republic of China; b School of Clinical Medicine, Hangzhou Medical College, Hangzhou, Zhejiang, People’s Republic of China; c Urology & Nephrology Center, Department of Nephrology, Zhejiang Provincial People’s Hospital (Affiliated People’s Hospital, Hangzhou Medical College), Hangzhou, Zhejiang, People’s Republic of China; d Department of Nephrology, the First Affiliated Hospital of Zhejiang Chinese Medical University (Zhejiang Provincial Hospital of Traditional Chinese Medicine), Hangzhou, Zhejiang, People’s Republic of China; e Department of Tuberculosis, Affiliated Hangzhou Chest Hospital, Zhejiang University School of Medicine, Hangzhou, Zhejiang, People’s Republic of China; f Department of Tuberculosis, Hangzhou Red Cross Hospital, Hangzhou, Zhejiang, People’s Republic of China; Rutgers New Jersey Medical School

**Keywords:** pulmonary tuberculosis, diabetes mellitus, metabolomic, transcriptome, NOTCH1

## Abstract

Pulmonary tuberculosis (PTB) and diabetes mellitus (DM) are common chronic diseases that threaten human health. Patients with DM are susceptible to PTB, an important factor that aggravates the complications of diabetes. However, the molecular regulatory mechanism underlying the susceptibility of patients with DM to PTB infection remains unknown. In this study, healthy subjects, patients with primary PTB, and patients with primary PTB complicated by DM were recruited according to inclusion and exclusion criteria. Peripheral whole blood was collected, and alteration profiles and potential molecular mechanisms were further analyzed using integrated bioinformatics analysis of metabolomics and transcriptomics. Transcriptional data revealed that lipocalin 2 (LCN2), defensin alpha 1 (DEFA1), peptidoglycan recognition protein 1 (PGLYRP1), and integrin subunit alpha 2b (ITGA2B) were significantly upregulated, while chloride intracellular channel 3 (CLIC3) was significantly downregulated in the group with PTB and DM (PTB_DM) in contrast to the healthy control (HC) group. Additionally, the interleukin 17 (IL-17), phosphatidylinositol 3-kinase (PI3K)-AKT, and peroxisome proliferator-activated receptor (PPAR) signaling pathways are important for PTB infection and regulation of PTB-complicated diabetes. Metabolomic data showed that glycerophospholipid metabolism, carbon metabolism, and fat digestion and absorption processes were enriched in the differential metabolic analysis. Finally, integrated analysis of both metabolomic and transcriptomic data indicated that the NOTCH1/JAK/STAT signaling pathway is important in PTB complicated by DM. In conclusion, PTB infection altered the transcriptional and metabolic profiles of patients with DM. Metabolomic and transcriptomic changes were highly correlated in PTB patients with DM. Peripheral metabolite levels may be used as biomarkers for PTB management in patients with DM.

**IMPORTANCE** The comorbidity of diabetes mellitus (DM) significantly increases the risk of tuberculosis infection and adverse tuberculosis treatment outcomes. Most previous studies have focused on the relationship between the effect of blood glucose control and the outcome of antituberculosis treatment in pulmonary tuberculosis (PTB) with DM (PTB_DM); however, early prediction and the underlying molecular mechanism of susceptibility to PTB infection in patients with DM remain unclear. In this study, transcriptome sequencing and untargeted metabolomics were performed to elucidate the key molecules and signaling pathways involved in PTB infection and the susceptibility of patients with diabetes to PTB. Our findings contribute to the development of vital diagnostic biomarkers for PTB or PTB_DM and provide a comprehensive understanding of molecular regulation during disease progression.

## INTRODUCTION

Tuberculosis (TB) is a chronic infectious disease caused by infection with Mycobacterium tuberculosis (1, 2). The World Health Organization reported that approximately 6.4 million people were newly diagnosed with TB and approximately 1.4 million died of TB in 2021 (3). TB can encroach on many organs of the human body, and pulmonary tuberculosis (PTB) is the most common consequence of TB infection in clinical practice (3). Diabetes mellitus (DM) is one of the most common chronic diseases that increases the risk of infection (4). The number of patients with active PTB aged 20 to 79 years was as high as 463 million in 2018, and this number is expected to increase to 700 million by 2045 owing to the aggravated immune environment in patients with DM (5). Patients with PTB and DM (PTB_DM) tend to have poor therapeutic efficacy, an increased risk of relapse, and a higher death rate (6, 7). Some studies have shown that the proportion of patients with DM among patients with PTB has been increasing, and the risk of treatment failure and death in patients with PTB_DM is 7.5 times and 6 times higher, respectively, than that in patients with PTB alone (8). Recently, most studies have focused on the relationship between the effect of blood glucose control and the outcome of anti-TB treatment in PTB_DM but lack timely prediction and deeper risk analysis of anti-TB failure. Therefore, the double burden of early prevention and treatment of PTB and DM has attracted increasing attention.

Many studies have reported that a high-glucose environment in patients with DM is conducive to the growth and reproduction of M. tuberculosis, leading to increased susceptibility to TB, a longer therapeutic cycle, and increased recurrence and mortality in PTB infection (9, 10). In addition, diabetes can result in disorganized glucose and lipid metabolism. Glycerol, a hydrolysis product of triglycerides, is an important carbon source for the growth and multiplication of M. tuberculosis and provides support for lung invasion (11). It has been suggested that an impaired immune response in diabetic patients may promote primary TB infection or reactivation of latent TB, thereby increasing both patient susceptibility to PTB and disease progression (12). A previous study revealed that diabetes increases the magnitude of gene expression changes in the transcriptome of patients with TB, with a particular increase in genes related to congenital inflammation and a decrease in those involved in the adaptive immune response (13). Compared with PTB patients, patients with diabetic TB and moderately hyperglycemic TB have a decreased type I interferon response (13, 14). Type 2 diabetes is associated with an increased frequency of mycobacterial antigen-specific CD4^+^ helper T cell type 1 (Th1) and type 17 (Th17), and PTB coexisting with type 2 diabetes is associated with changes in CD8^+^ T and natural killer (NK) cells that produce cytokines and express cytotoxic molecules, leading to aggressive abnormalities in pathology (15, 16). However, the underlying cause and mechanism of susceptibility to PTB in patients with diabetes are yet to be elucidated in a holistic manner. The current diagnostic approach for PTB_DM relies mainly on typical clinical symptoms, imaging findings, laboratory tests, and glycosylated hemoglobin/blood glucose monitoring (17). However, an increasing number of cases of PTB lack typical clinical symptoms and specific imaging findings, making laboratory tests such as acid-fast-stained sputum smears, sputum cultivation, Xpert MTB/RIF, and T-SPOT indispensable for the diagnosis of TB (18). As the detection rate of sputum smears is low and the period of sputum culture is long, imaging analysis can easily cause misdiagnosis owing to a lack of specificity. More importantly, conventional diagnostic methods are not available for predicting the risk and predisposing factors for PTB at early disease stages. Therefore, it is urgent to discover more effective tools.

Integrated analysis of metabolomics and transcriptomics has become an effective tool for understanding the disease development process, uncovering regulatory mechanisms and key factors, and determining the relationships between genes and metabolites and the underlying complex molecular networks. In this study, we recruited 39 clinical subjects, comprising healthy subjects and patients newly diagnosed with PTB, with or without DM. We compared and combined transcriptome sequencing and metabolome profiling data to elucidate the molecules and pathways associated with PTB infection and the susceptibility of patients with diabetes to PTB. This study facilitates the exploration of critical diagnostic biomarkers of PTB or PTB concomitant with diabetes and provides a comprehensive understanding of the molecular responses involved.

## RESULTS

### Imaging characteristics of different groups.

**FIG 1:**
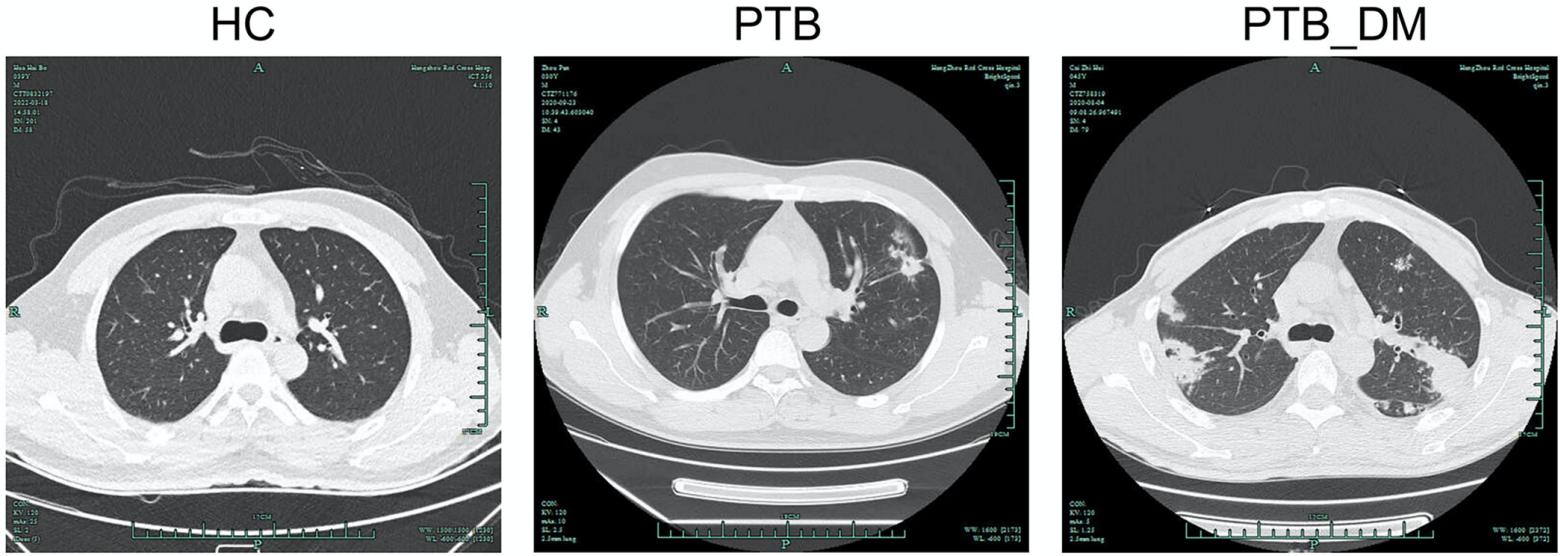
Representative lung CT images from the HC, PTB, and PTB_DM groups.

In total, 13 healthy control (HC) patients, 13 pulmonary tuberculosis (PTB) patients, and 13 patients with pulmonary tuberculosis with diabetes mellitus (PTB_DM) were recruited. Whole-blood and serum samples from the included patients were collected and stored in an ultralow-temperature freezer. Clinical information was statistically analyzed, as shown in Table 1. Compared to the HC group, the positivity rates of the tuberculin skin test, gamma interferon release, GeneXpert MTB/RIF, imaging features of TB, sputum culture, sputum smear, and cough for >2 weeks in patients with PTB and PTB_DM were significantly higher (Table 1). In the PTB_DM group, the incidence of fever for >2 weeks, hemoglobin A1c, and high blood glucose levels were significantly higher than those in the other groups. Compared with the PTB group, the positive result rates of GeneXpert MTB/RIF, imaging features of TB, sputum culture, fever for >2 weeks, hemoglobin A1c, hemoglobin blood glucose, and diabetes tests were significantly higher in the PTB_DM group. Representative computed tomography (CT) images of the subjects in each group are shown in Fig. 1. Compared with the normal lung images of the HC group, small centrilobular nodules were observed in the left lungs of patients in the PTB group, and nodules with cavitation were observed in both lungs of patients in the PTB_DM group (Fig. 1). These data indicate that poor blood glucose maintenance in patients with diabetes may aggravate the symptoms and disease progression of PTB.

**TABLE 1:** Clinical information collected from the candidates

Parameter	Value for group^*a*^		
	Health (*n* = 13)	Pulmonary tuberculosis (*n* = 13)	Pulmonary tuberculosis with type 2 diabetes (*n* = 13)
Age (yrs)	40.2 ± 6.6	31.4 ± 11.0	50.9 ± 11.9
Sex (male/female)	4/9	10/3	11/2
Body mass index (kg/m^2^)	23.7 ± 3.1	19.6 ± 2.3	23.9 ± 2.7
Hypertension (yes/no)	0/13	0/13	0/13
Tuberculin skin test (positive/negative)	0/13	11/2**	13/0**
Gamma interferon release assay (positive/negative)	0/13	10/3**	13/0**
GeneXpert MTB/RIF (positive/negative)	0/13	9/4**	13/0**^^^
Imaging features of tuberculosis lesion (positive/negative)	0/13	13/0**	13/0**^^^^
Sputum culture positive (positive/negative)	0/13	9/4**	13/0**^^^
Sputum smear positive (positive/negative)	0/13	7/6**	13/0**
Cough for >2 wks (yes/no)	0/13	12/1**	12/1**
Fever for >2 wks (yes/no)	0/13	1/12	2/11**
Night sweats for >2 wks (yes/no)	0/13	0/13	2/11
Whether situation was improved by antituberculosis treatment (yes/no)		13/0	13/0
History of tuberculosis (yes/no)	0/13	0/13	0/13
Diabetes (yes/no)	0/13	0/13	13/0**^^^^
Glycosylated hemoglobin A1c (%)	4.9 ± 0.7	4.4 ± 0.7	10 ± 2.6**^^^^
Blood glucose (mmol/L)	5.3 ± 0.4	5.7 ± 1.1	12.4 ± 8.2**^^^^
24-h urinary protein quantification (g/day)			0.13 ± 0.0
Urinary albumin-to-creatinine ratio			11.1 ± 4.8
Endogenous creatinine clearance rate (mL/min)			80.3 ± 16.8
Total protein (g/L)	75.5 ± 3.1	72.2 ± 7.0	71.9 ± 6.7
White blood cells (10^9^/L)	6.1 ± 1.6	6.7 ± 1.6	6.0 ± 2.6
Red blood cells (10^12^/L)	4.9 ± 0.7	4.6 ± 0.4	4.6 ± 0.8
Hemoglobin (g/L)	134.4 ± 16.1	138.1 ± 14.2	129.5 ± 19.7
Platelets (10^9^/L)	279.2 ± 82.4	279.5 ± 100.8	233.4 ± 67.8
Creatinine (μmol/L)	75.21 ± 11.1	76.2 ± 15.3	80.5 ± 18.5
High-density lipoprotein (mmol/L)	1.5 ± 0.3	1.3 ± 0.8	1.0 ± 0.1
Low-density lipoprotein (mmol/L)	2.84 ± 0.6	2.4 ± 0.6	2.8 ± 0.5

a The single caret means that we did not perform these detection during hospitalization. **, *P *< 0.01 versus health; ^^^^, *P *< 0.01 versus pulmonary tuberculosis.

### Sequencing data and repeated correlation assessment.

**FIG 2:**
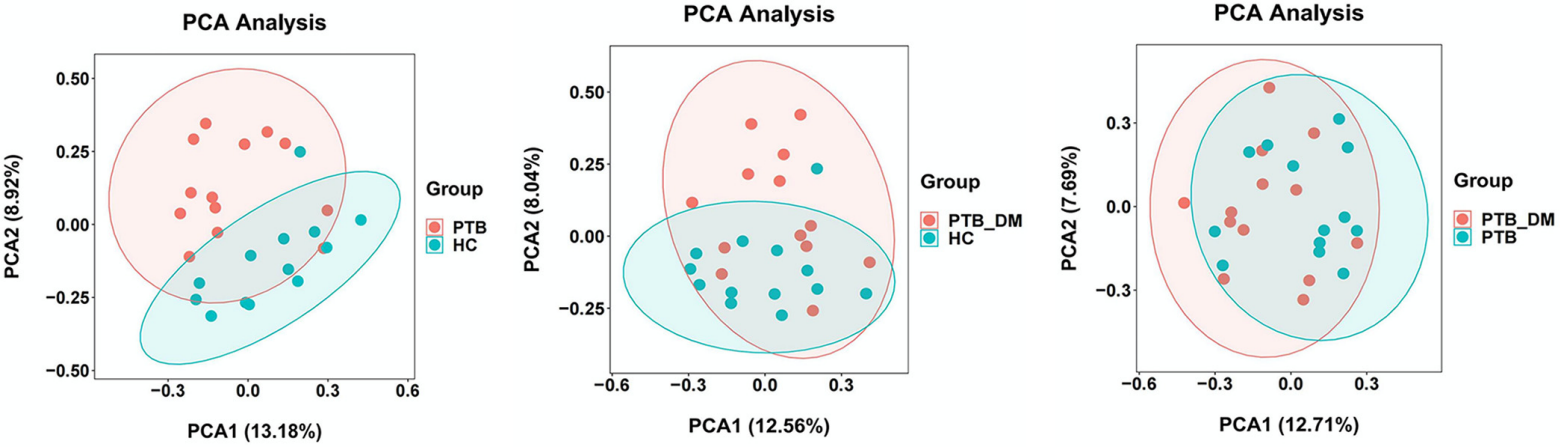
Pairwise PCA of the three groups.

The transcriptome data from the 39 samples yielded 253.36 GB of valid data. The Q20 and Q30 values were greater than 99.9% and 98%, respectively. The efficiency of valid read alignment with the human reference genome varied from 94.94% to 98.64% among the samples (Table 2). Subsequent gene expression analyses were performed based on the sequencing results. The correlation analysis of samples facilitates their clustering, which can be explored using principal-component analysis (PCA) to help understand the repeatability between samples. As shown in Fig. 2, the degree of dispersion was highest between the PTB and HC groups, followed by that of the PTB_DM versus HC group. In the PTB_DM and PTB groups, the degree of dispersion of each component was similar (Fig. 2).

**TABLE 2:** Sequencing data of transcriptome^*a*^

Sample	Raw data		Valid data		Valid ratio (reads)	Q20 (%)	Q30 (%)	GC content (%)
	No. of reads	No. of bases	No. of reads	No. of bases				
HC_1	51,764,970	7.76G	50,489,426	7.57G	97.54	99.99	98.73	56
HC_2	52,924,160	7.94G	51,845,568	7.78G	97.96	99.99	98.72	56.5
HC_3	53,225,044	7.98G	52,129,860	7.82G	97.94	99.98	98.7	57
HC_4	48,606,914	7.29G	47,947,524	7.19G	98.64	99.99	98.84	59
HC_5	51,518,346	7.73G	50,388,846	7.56G	97.81	99.98	98.84	57
HC_6	35,822,648	5.37G	34,930,862	5.24G	97.51	99.98	98.84	57.5
HC_7	34,086,100	5.11G	33,152,176	4.97G	97.26	99.97	98.77	55
HC_8	33,349,492	5.00G	32,297,930	4.84G	96.85	99.97	98.84	56
HC_9	48,125,052	7.22G	46,918,656	7.04G	97.49	99.98	98.82	56.5
HC_10	45,760,888	6.86G	44,453,974	6.67G	97.14	99.98	98.74	56.5
HC_11	51,068,956	7.66G	49,615,576	7.44G	97.15	99.98	98.77	55
HC_12	55,099,484	8.26G	53,983,558	8.10G	97.97	99.98	98.87	56
HC_13	39,696,614	5.95G	38,515,882	5.78G	97.03	99.98	97.43	55
PTB_1	51,456,786	7.72G	50,037,224	7.51G	97.24	99.99	98.77	53.5
PTB_2	53,252,694	7.99G	52,112,220	7.82G	97.86	99.98	98.75	54
PTB_3	53,753,072	8.06G	52,748,704	7.91G	98.13	99.99	98.82	55.5
PTB_4	51,445,324	7.72G	49,945,388	7.49G	97.08	99.98	98.62	55.5
PTB_5	50,874,034	7.63G	49,741,656	7.46G	97.77	99.98	98.62	55
PTB_6	43,531,036	6.53G	42,661,896	6.40G	98	99.98	98.58	56
PTB_7	47,277,002	7.09G	45,830,542	6.87G	96.94	99.98	98.82	57.5
PTB_8	43,236,406	6.49G	41,338,380	6.20G	95.61	99.98	98.56	58
PTB_9	49,943,986	7.49G	48,523,664	7.28G	97.16	99.98	98.63	54
PTB_10	41,439,916	6.22G	39,952,034	5.99G	96.41	99.97	98.38	53.5
PTB_11	41,979,590	6.30G	40,764,086	6.11G	97.1	99.98	98.5	56.5
PTB_12	38,121,674	5.72G	37,110,986	5.57G	97.35	99.98	98.61	54
PTB_13	49,400,904	7.41G	48,415,242	7.26G	98	99.98	98.76	55.5
PTB_DM_1	43,858,142	6.58G	42,983,038	6.45G	98	99.99	98.72	54.5
PTB_DM_2	38,288,004	5.74G	37,202,390	5.58G	97.16	99.98	98.45	57
PTB_DM_3	50,768,778	7.62G	49,395,224	7.41G	97.29	99.98	98.73	56.5
PTB_DM_4	49,466,268	7.42G	46,961,066	7.04G	94.94	99.98	98.84	57.5
PTB_DM_5	46,297,056	6.94G	45,307,932	6.80G	97.86	99.98	98.68	58
PTB_DM_6	44,979,746	6.75G	43,801,618	6.57G	97.38	99.98	98.8	56
PTB_DM_7	43,956,478	6.59G	42,852,516	6.43G	97.49	99.98	98.91	57
PTB_DM_8	41,701,824	6.26G	39,708,022	5.96G	95.22	99.97	98.56	54.5
PTB_DM_9	40,273,818	6.04G	39,093,862	5.86G	97.07	99.98	98.59	53
PTB_DM_10	33,719,898	5.06G	32,888,340	4.93G	97.53	99.98	98.83	57.5
PTB_DM_11	34,254,052	5.14G	33,267,354	4.99G	97.12	99.97	98.66	56
PTB_DM_12	52,412,698	7.86G	49,823,722	7.47G	95.06	99.98	98.88	56
PTB_DM_13	53,569,396	8.04G	51,814,084	7.77G	96.72	99.97	98.62	54

a G indicates a Computer storage unit (Gigabyte).

### RNA-Seq analysis revealed different expression patterns in each group.

**FIG 3:**
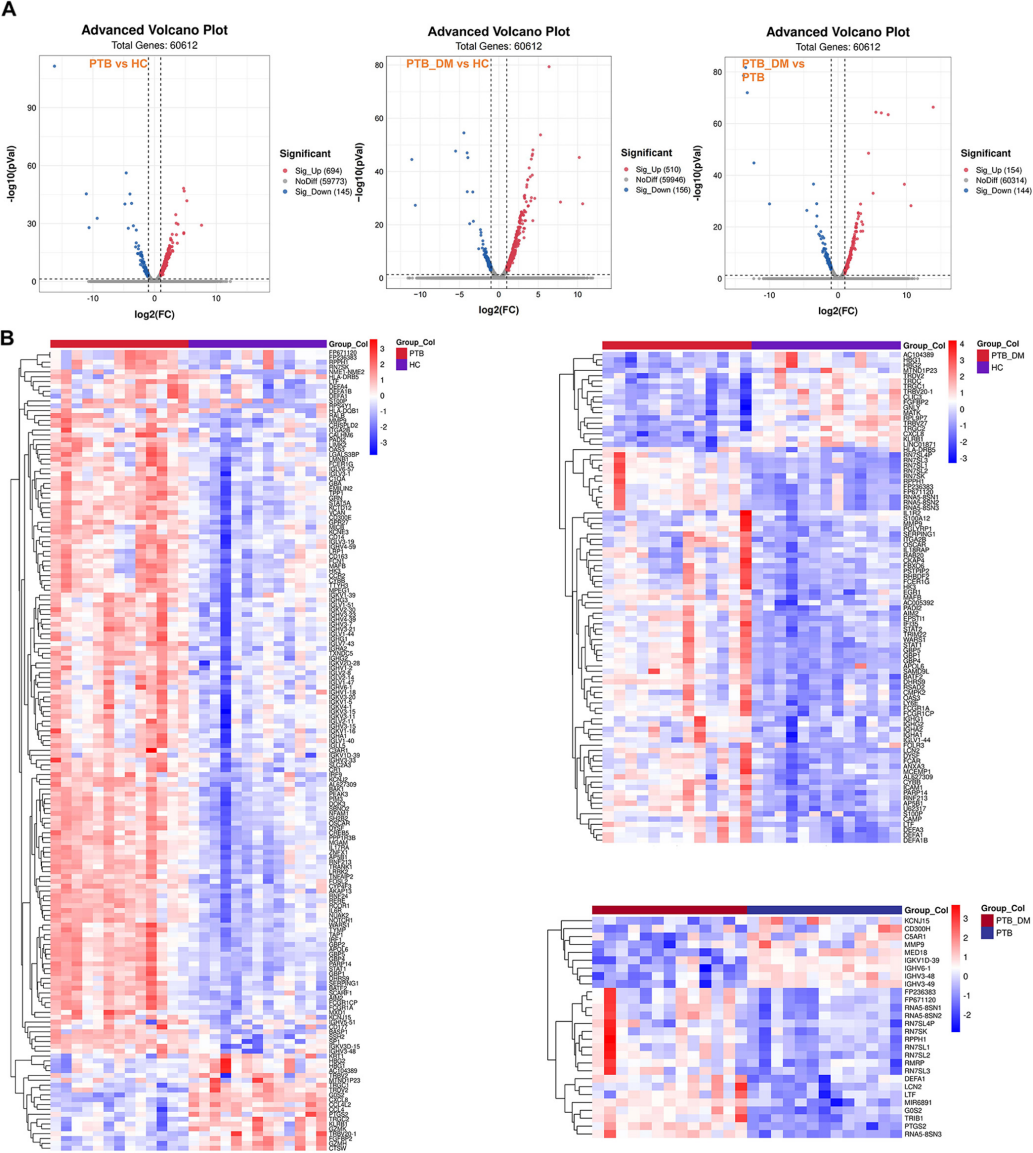
Pairwise differential analysis of the three groups. (A) Volcano plot of PTB versus HC groups, PTB_DM versus HC groups, and PTB_DM versus PTB groups. FC ≥ 2 or FC ≤ 0.5; *P* < 0.05. (B) Clustered heat map of PTB versus HC groups, PTB_DM versus HC groups, and PTB_DM versus PTB groups (color scale indicates the fold changes of DEGs).

The term differentially expressed genes (DEGs) refers to significantly upregulated and downregulated genes among the samples. The genes were screened according to the combined threshold values of differential fold change (FC ≥ 2 or FC ≤ 0.5) and significance (*P < *0.05). The volcano plots in Fig. 3A indicates the expression profiles of DEGs, as judged by the specified standard. There were 649 significantly upregulated and 145 significantly downregulated genes as determined by comparison of the PTB and HC groups. When we compared the PTB_DM group to the HC group, we observed that 156 genes were significantly downregulated and 510 genes were upregulated. In the PTB_DM versus PTB comparison, the number of significantly upregulated genes was reduced to 154, and 144 downregulated genes were observed (Fig. 3A). To directly visualize the expression profiles of the different groups, a clustered heat map was applied to analyze the DEGs according to the refined threshold values (FC ≥ 2 or FC ≤ 0.5; *P < *0.05; fragments per kilobase per million [FPKM] ≥ 10) (Fig. 3B). Transcriptome sequencing RNA-seq sequencing (RNA-Seq) data revealed that in contrast to the HC group, DM patients with PTB infection exhibited a different expression profile from that of patients with PTB infection only. As presented in Table 3, basic leucine zipper ATF-like transcription factor 2 (BATF2), polycythemia rubra vera receptor 1 (PRV1 or CD177), and IgG1 heavy chain constant region (IGHG1) were significantly upregulated, while G_0_/G_1_ switch gene 2 (G0S2), hemoglobin subunit gamma 1 (HBG1), and hemoglobin subunit gamma 2 (HBG2) were significantly downregulated in PTB patients compared to the HC group (Table 3). Expressions of lipocalin 2 (LCN2), defensin alpha 1 (DEFA1), peptidoglycan recognition protein 1 (PGLYRP1), and integrin subunit alpha 2b (ITGA2B) were significantly upregulated, while chloride intracellular channel 3 (CLIC3) was significantly downregulated in the PTB_DM group in contrast to the HC group (Table 3). Possibly, these DEGs participated in the processes of PTB and PTB_DM diseases.

**TABLE 3:** Potential biomarkers screened by transcriptomics

Gene name	Gene identifier	*P* value	*Q* value	Fold change	Regulation	Significant
BATF2	ENSG00000168062	4.67801E−23	2.99047E−20	6.11 (PTB/HC)	Up	Yes
CD177	ENSG00000204936	3.98164E−15	1.0836E−12	3.93 (PTB/HC)	Up	Yes
IGHG1	ENSG00000211896	2.75736E−14	6.33461E−12	3.88 (PTB/HC)	Up	Yes
G0S2	ENSG00000123689	1.54416E−14	3.72194E−12	0.23 (PTB/HC)	Down	Yes
HBG1	ENSG00000213934	5.09381E−46	1.24824E−42	0.06 (PTB/HC)	Down	Yes
HBG2	ENSG00000196565	6.08075E−57	4.47027E−53	0.04 (PTB/HC)	Down	Yes
LCN2	ENSG00000148346	2.72086E−24	8.32357E−22	6.89 (PTB_DM/HC)	Up	Yes
DEFA1	ENSG00000206047	1.13861E−15	1.72365E−13	4.63 (PTB_DM/HC)	Up	Yes
PGLYRP1	ENSG00000008438	1.56795E−12	1.79873E−10	3.65 (PTB_DM/HC)	Up	Yes
ITGA2B	ENSG00000005961	1.66286E−08	1.12007E−06	2.80 (PTB_DM/HC)	Up	Yes
CLIC3	ENSG00000169583	4.53652E−06	0.000178591	0.45 (PTB_DM/HC)	Down	Yes

### Functional enrichment analysis of DEGs.

**FIG 4:**
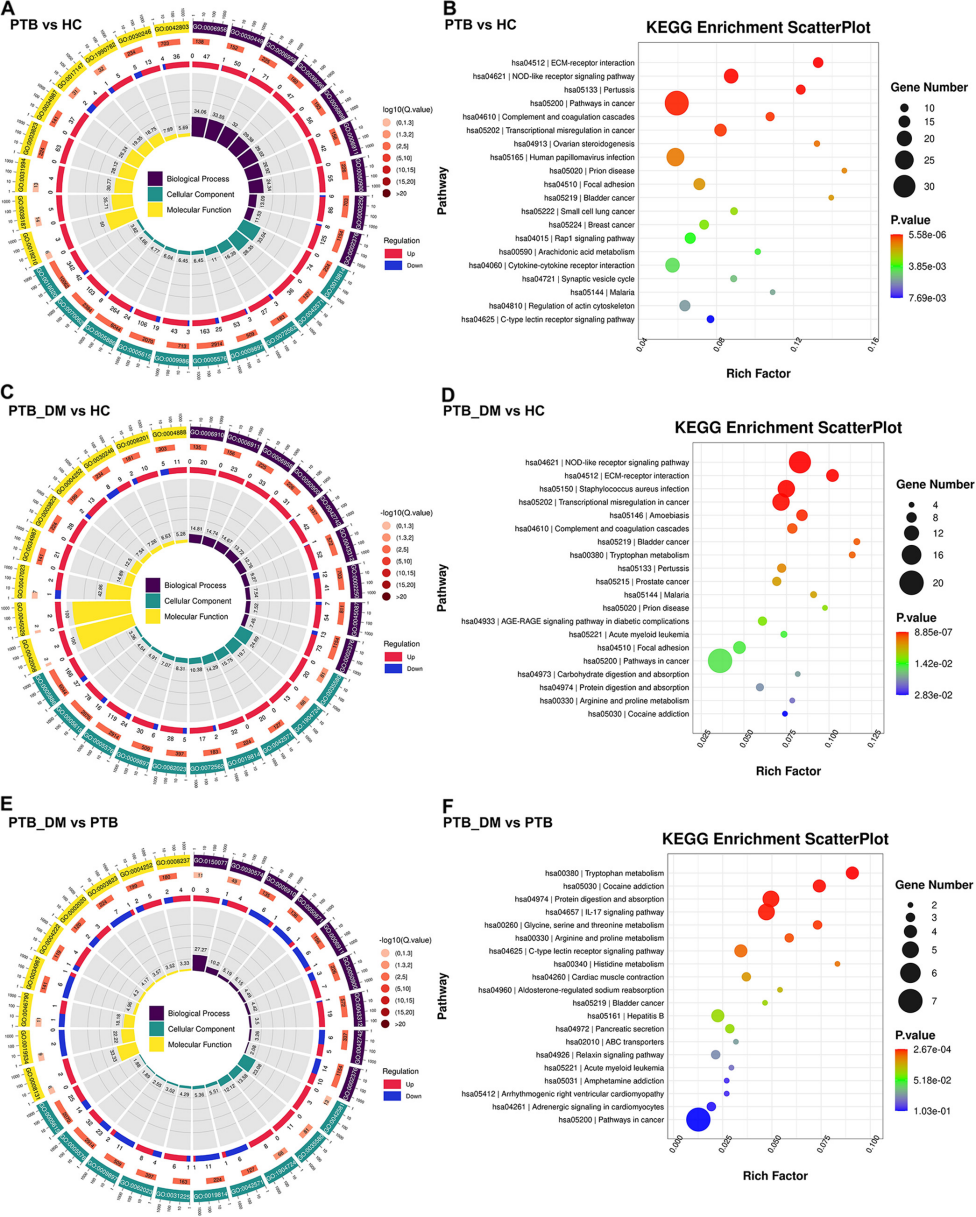
Functional enrichment of DEGs. (A, C, and E) GO enrichment of PTB versus HC, PTB_DM versus HC, and PTB_DM versus PTB groups. (B, D, and F) KEGG enrichment of PTB versus HC, PTB_DM versus HC, and PTB_DM versus PTB groups.

To gain further insight into the underlying mechanisms and pathways involved in the influence of blood glucose on PTB infection, we performed functional enrichment analysis of DEGs from different groups. The Gene Ontology (GO) and Kyoto Encyclopedia of Genes and Genomes (KEGG) databases were used to perform functional enrichment analysis of DEGs in the PTB versus HC, PTB_DM versus HC, and PTB_DM versus PTB groups (Fig. 4). In the PTB versus HC groups, the KEGG enrichment analysis results showed that extracellular matrix (ECM)-receptor interaction, NOD-like receptor signaling pathway, and pertussis were the top three enriched pathways (Fig. 4B). Compared to those in the HC group, the DEGs in the PTB_DM group were mostly enriched in the NOD-like receptor signaling pathway, ECM-receptor interaction, and Staphylococcus aureus infection pathways (Fig. 4D). DEGs in the PTB_DM versus PTB group comparison were mostly enriched in tryptophan metabolism, cocaine addiction, and protein digestion and absorption pathways (Fig. 4F). These results indicate that metabolic and immune pathway regulation may play a role in PTB infection in patients with DM.

### Quality control (QC) and sample correlation analysis of metabolomics data.

**FIG 5:**
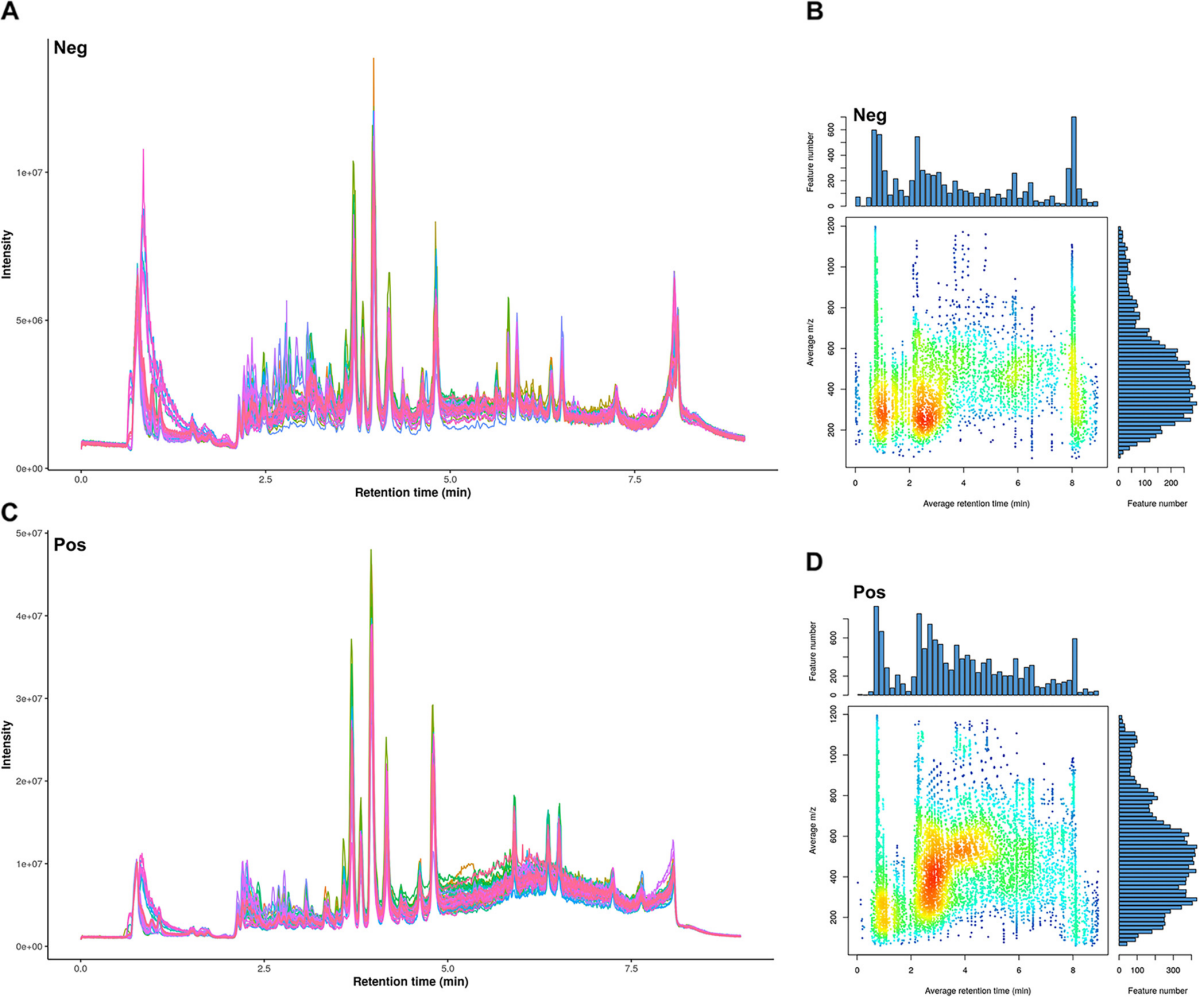
Quality control of metabolomics analyses. (A and C) The total ion chromatogram (TIC) exhibits an integrated view of the mass spectrum signal intensity of the samples in both positive- and negative-ion modes. (B and D) *m/z*-RT distribution of metabolites in both positive- and negative-ion modes. Each dot represents a substance, and the color indicates the density of the substance in that area: brighter colors indicate higher feature densities.

**FIG 6:**
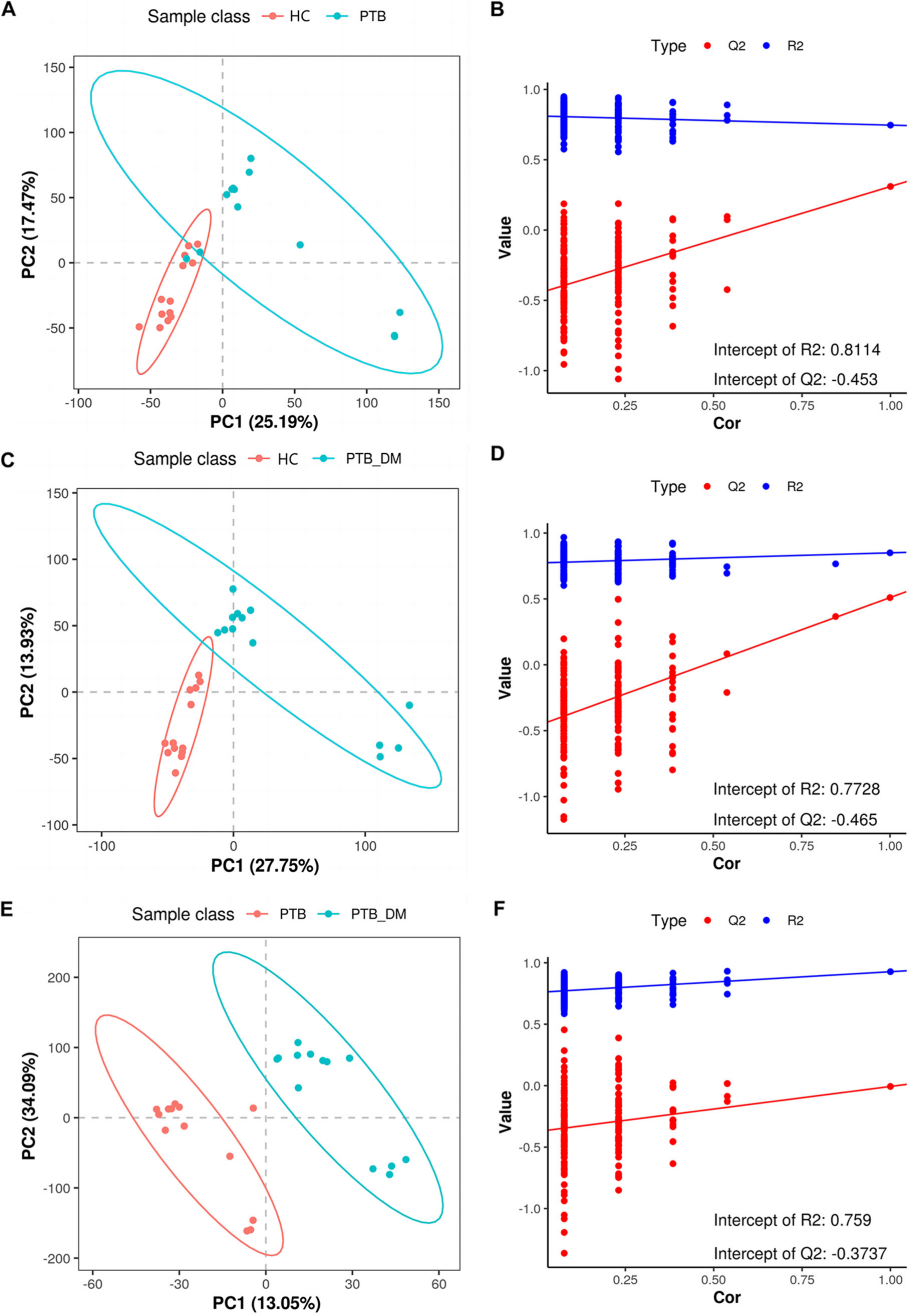
PLS-DA plot comparison of metabolite profiles. (A) PLS-DA plot of the PTB and HC groups. (B) Validation of the PTB and HC PLS-DA model using the 200-time permutation test. (C) PLS-DA plot of the PTB_DM and HC groups. (D) Validation of the PTB_DM and HC PLS-DA model using the 200-time permutation test. (E) PLS-DA plot of the PTB_DM and PTB groups. (F) Validation of the PTB_DM and PTB PLS-DA model using the 200-time permutation test.

To answer the question of how metabolic pathways are involved in PTB infection in patients with DM, we performed liquid chromatography-mass spectrometry (LC-MS) to analyze the changes in the metabolomics of patients with PTB_DM. The total ion chromatogram (TIC) exhibited an integrated view of the mass spectrum signal intensity of the samples, reflecting all the metabolites separated by liquid chromatography (Fig. 5A and C). Based on the TIC mapping in the positive- and negative-ion modes, we observed that the spectrograms of multiple samples overlapped well, and the retention time and peak response intensity fluctuated only slightly (Fig. 5), indicating that the MS instrument performed as normal for the entire duration of sample testing. We further developed a supervised partial least-squares discriminant analysis (PLS-DA) methodological model to better account for the changes in metabolites between each comparison group (Fig. 6A, C, and E). A permutation test of 200 random numbers was used to verify the PLS-DA model. The results showed that all red predictability (Q2) values to the left were lower than the original points to the right (Fig. 6B, D, and F), proving that the PLS-DA model was effective and stable. Based on the PLS-DA model, there was a distinct difference between the compared groups, indicating a distinct metabolomic profile in each group. In addition, the PLS-DA score plots of the two groups showed apparent differences between the PTB versus HC, PTB_DM versus HC, and PTB_DM versus PTB groups. As presented in the PLS-DA permutation test plots, the goodness of fit (R2Y) and Q2 values were as follows: R2Y = 0.8114 and Q2 = −0.453 in PTB versus HC, R2Y = 0.7728 and Q2 = −0.465 in PTB_DM versus HC, and R2Y = 0.759 and Q2 = −0.3737 in the PTB_DM versus PTB groups (Fig. 6B, D, and F). Based on the PLS-DA model, the prediction model, repeatability, and differences between the two groups analyzed were reliable and stable.

### Analysis of differential metabolites.

**FIG 7:**
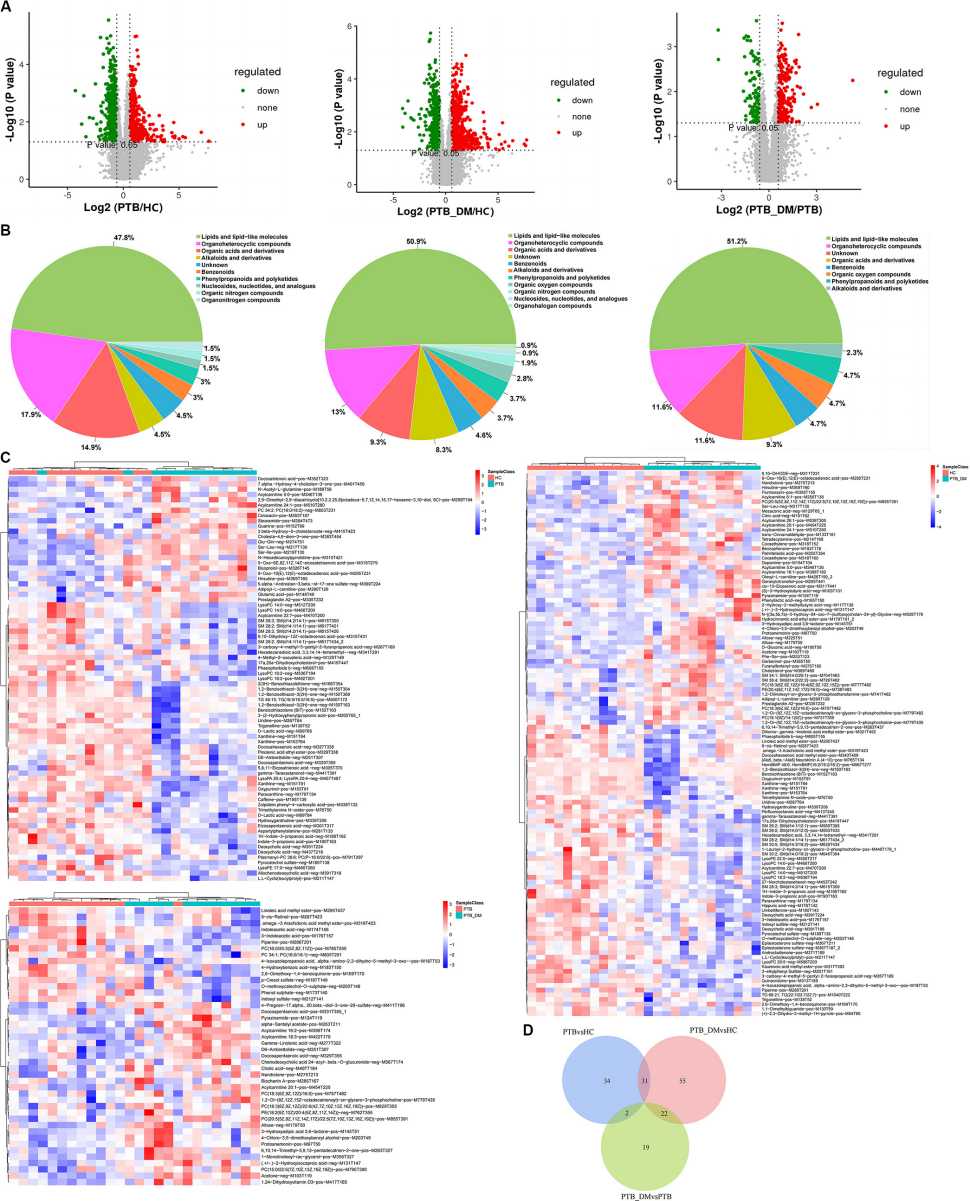
Functional enrichment of differential metabolites. (A) Volcano maps of different metabolites in the three groups. (B) Distribution of different metabolite superclasses in the three groups. (C) Heat maps of different metabolites. VIP ≥ 1, |fold change| > 1.2, and *P* < 0.05. (D) Venn diagram of the different metabolites in the three groups.

In total, 1,116 metabolites were identified in the three groups of 39 samples using a combination of positive- and negative-ion modes in mass spectrometry (see Table S1 in the supplemental material). The numbers of metabolites identified in the positive- and negative-ion modes were 758 and 358, respectively. Volcano plots were constructed to determine the differences in the metabolites among the three groups. As shown in Fig. 7A, 130 metabolites showed visible alterations; 92 metabolites were upregulated, 38 metabolites were downregulated, and most of the other metabolites were not significantly altered. The proportions of the metabolites according to their chemical taxonomy are shown in Fig. 7B. The top three differential metabolites, including lipid molecules, organoheterocyclic compounds, and organic acids, accounted for 80.6%, 73.2%, and 74.4% in the PTB versus HC, PTB_DM versus HC, and 74.4% in PTB_DM versus PTB groups, respectively (Fig. 7B). To visualize the overall alteration of the serum metabolome, clustered heatmaps of metabolites were generated with the screening conditions of variable importance in the projection (VIP) of ≥1, |fold change| of >1.2, and *P* value of <0.05 (Fig. 7C). The resultant heat map of PTB versus HC indicated dramatic changes in the metabolome, the top metabolites of which included *N*-acetyl-l-glutamine, 2,9-dimethyl-2,9-diazatricyclo[10.2.2.25,8]octadeca-5,7,12,14,15,17-h, glutamic acid, 5-oxo-6E,8Z,11Z,14Z-eicosatetraenoic acid, indole-3-propanoic acid, caffeine, paraxanthine, pyrocatechol sulfate, trimethylamine *N*-oxide, and xanthine. Metabolites such as citric acid, *N*-[(3a,5b,7a)-3-hydroxy-24-oxo-7-(sulfooxy)cholan-24-yl]-glycine (19, 20), hydrocinnamic acid ethyl ester, (*S*)-3-gydroxybutyric acid, benzophenone, paraxanthine, hippuric acid, pyrocatechol sulfate, and indole-3-propionic acid exhibited the most significant changes in the PTB_DM group compared to the PTB group. Furthermore, the number of significantly different metabolites was less than in PTB versus HC or PTB_DM versus HC, consisting of chenodeoxycholic acid 24-acyl-d-glucuronide, PE(18:2(9Z,12Z)/20:4(5Z,8Z,11Z,14Z)), cholic acid, 3-indoleacetic acid, 4-hydroxybenzoic acid, 2,6-dimethoxy-1,4-benzoquinone, 4-isoxazolepropanoic acid, indoxyl sulfate, and piperine. A Venn diagram of the different metabolites in each comparison is shown in Fig. 7D. Thirty-one shared differential metabolites were found in the PTB versus HC and PTB_DM versus HC comparisons, and 24 DM-associated differential metabolites (PTB_DM versus PTB) were also associated with PTB (Fig. 7D). Our results revealed PTB-specific DM-associated differential metabolites in the three comparisons.

### Functional enrichment analysis of differential metabolites.

**FIG 8:**
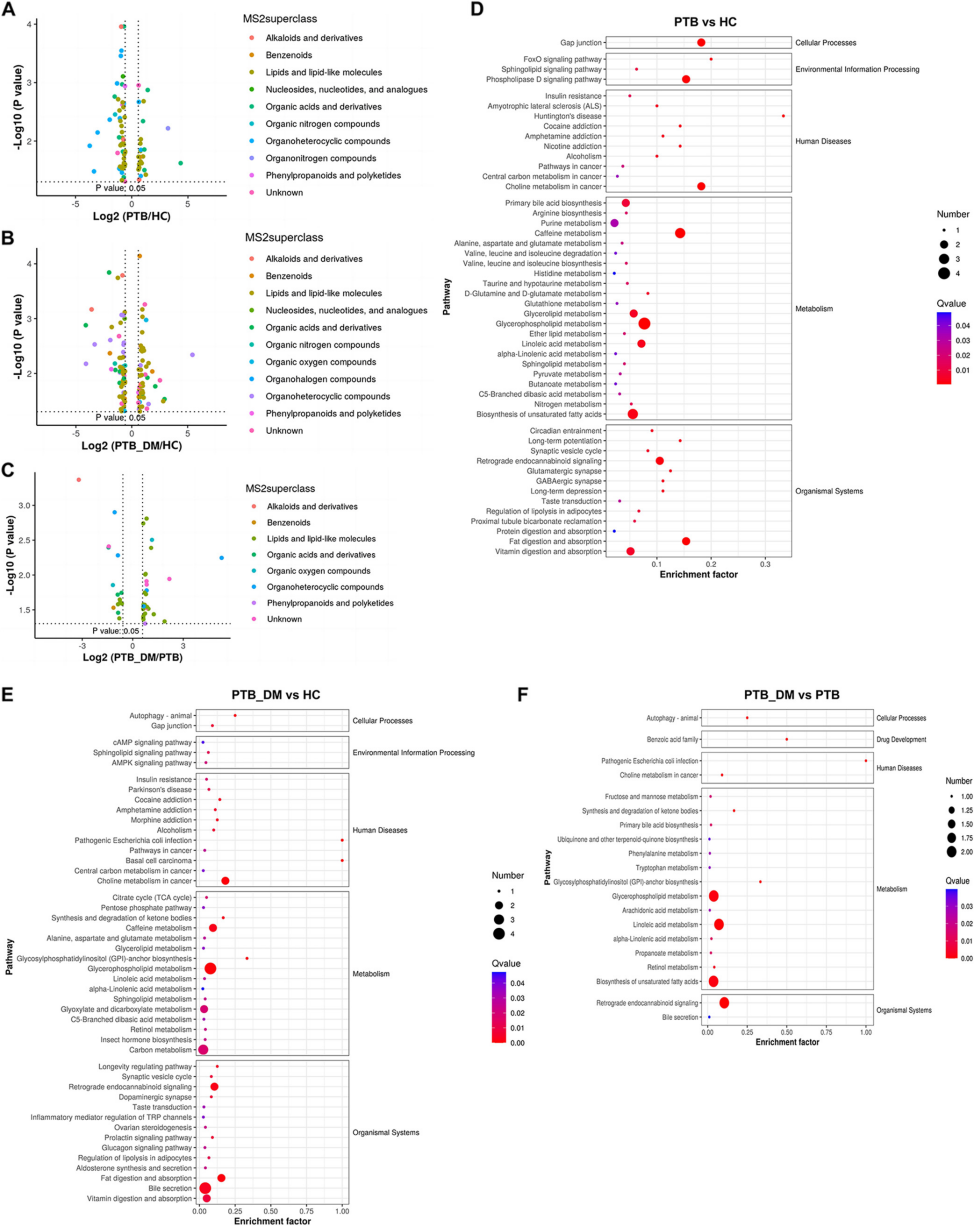
MS2 superclass and enrichment of different metabolites. (A to C) Scatterplots of different metabolites in the MS2 superclass. (D to F) KEGG analysis of PTB versus HC, PTB_DM versus HC, and PTB_DM versus PTB. *P* < 0.05.

In PTB versus HC, metabolites were enriched in 49 pathways, most notably glycerophospholipid metabolism, caffeine metabolism, biosynthesis of unsaturated fatty acids, glycerolipid metabolism, fat digestion and absorption, purine metabolism, vitamin digestion and absorption, choline metabolism in cancer, and retrograde endocannabinoid signaling (Fig. 8A and D). In PTB_DM versus HC, metabolites were mapped to 46 metabolic pathways, exhibiting enrichment in choline metabolism in cancer, glycerophospholipid metabolism, carbon metabolism, fat digestion and absorption, bile secretion, caffeine metabolism, retrograde endocannabinoid signaling, and glyoxylate and dicarboxylate metabolism (Fig. 8B and E). In PTB_DM versus PTB, metabolites were mapped to 49 metabolic pathways, including those enriched in retrograde endocannabinoid signaling, biosynthesis of unsaturated fatty acids, glycerophospholipid metabolism, and linoleic acid metabolism (Fig. 8C and F). The shared pathways associated with differentially expressed metabolites and transcripts in all three comparisons (*P* < 0.05) were retrograde endocannabinoid signaling and glycerophospholipid metabolism. The other four major pathways between PTB versus HC and PTB_DM versus HC were caffeine metabolism, fat digestion and absorption, vitamin digestion and absorption, and choline metabolism in cancer (Fig. 4 and 8). Using functional enrichment analysis, we identified the shared differential metabolic pathways.

### Combined transcriptomics and metabolomics analysis.

**FIG 9:**
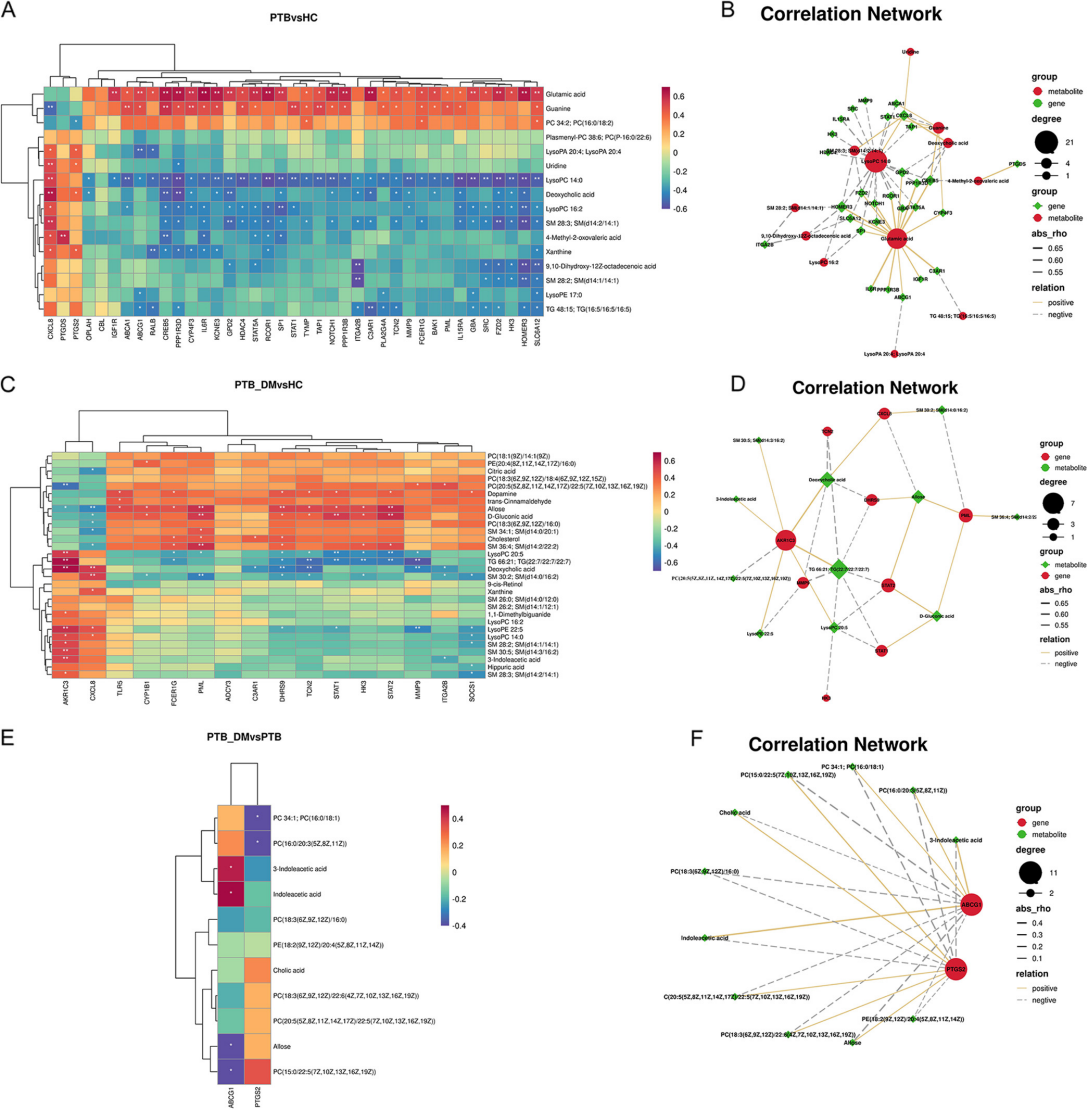
Spearman correlation analysis of transcripts and metabolites. (A, C, and E) Correlation heat maps of transcripts and metabolites (*, *P* < 0.05; **, *P* < 0.01). Results are expressed as means ± SEMs. (B, D, and F) Correlation network analyses of transcripts and metabolites (solid line, positive correlation; dashed line, negative correlation; thick line, significant correlation).

To avoid analysis bias from metabolomics or transcriptomics alone, transcripts were identified based on targeted metabolites in the KEGG database, both of which were then mapped to the related joint pathways. Then, Spearman correlation analysis was performed to calculate possible correlations between differential metabolites and differential genes in the three comparison groups (correlation coefficient *R* > 0.8, significance test *P* < 0.05) (Fig. 9A, C, and E). As shown in Fig. 9B, a heat map and network map were applied to understand the remarkable regulatory relationships between the three groups. In the PTB versus HC groups, the transcripts significantly related to the targeted metabolome were highly concentrated in lysoPC14:0 and glutamic acid. LysoPC14:0 was negatively correlated with most of the screened genes; however, shared genes such as *NOTCH1*, *STAT5A*, *HOMER3*, *SLC6A12*, *SP1*, *KCNE3*, and *FZD2* were positively correlated with glutamic acid (Fig. 9B). In the PTB_DM versus HC groups, genes such as *MMP9*, *AKR1C3*, *STAT1*, *STAT2*, *DHRS9*, and *TCN2* were related to the triacylglycerol TG66:21. *AKR1C3*, which exhibited a high degree score, may act as a major regulator. These genes were associated with many other important metabolites, including 3-indoleacetic acid, deoxycholic acid, and lysoPE22:5 (Fig. 9D). In the PTB_DM versus HC groups, *PTGS2* and *ABCG1* exhibited opposite roles in one-to-many associations with metabolites, such as 3-indoleacetic acid, indoleacetic acid, cholic acid, allose, and PC34:1 (Fig. 9F). The integration of metabolomics and transcriptomics analyses revealed important metabolites and pathways involved in PTB infection in patients with DM.

## DISCUSSION

PTB infection remains a major threat to health, and an increasing number of studies are focusing on discovering clinical biomarkers of PTB progression because the detection rate using sputum smears is low (21, 22). Recently, some studies have reported that patients with PTB and DM exhibit more severe PTB infection (23). In this study, transcriptional and metabolic profiling revealed the underlying regulatory pathways and metabolites in PTB-infected patients with DM. From our combined analysis, we determined that the NOTCH1 and STAT family genes were mostly enriched in both transcriptional and metabolic data. Therefore, we predicted that the NOTCH1/STAT pathway is an important pathway in regulating PTB infection progression. Some studies have also reported this regulatory pathway to be involved in the PTB immune response (24). Further studies are required to confirm the regulatory mechanisms of the NOTCH1/STAT pathway in PTB.

First, we performed transcriptional profiling to compare the PTB and HC, PTB_DM and HC, and PTB_DM and PTB groups. Differential expression analysis revealed that metabolic pathways were the major enriched pathways, indicating that PTB infection aggravates metabolism during disease progression. Top enriched pathways, such as the interleukin 17 (IL-17), phosphatidylinositol 3-kinase (PI3K)-AKT, and peroxisome proliferator-activated receptor (PPAR) signaling pathways, have recently been reported to contribute to PTB infection progression and glucose metabolism (19, 20, 25, 26). For example, IL-17 is one of the Th17-related cytokines, and the IL-17 signaling pathway is dysregulated or disrupted in patients with TB (19). The PI3K-AKT-mTOR signaling pathway is inhibited in patients with active TB and induces FOXP3^+^ regulatory T cell proliferation (20). The PPAR signaling pathway is highly expressed in alveolar macrophages, and M. tuberculosis differentially regulates Mcl-1 and Bax expression through PPAR to limit apoptosis (26). These data led us to compare the transcriptomes of PTB-infected patients with DM to those of patients with PTB infection only. In addition to the IL-17 signaling pathway, metabolic pathways such as tryptophan metabolism, histidine metabolism, and arginine and proline metabolism were significantly enriched in the DEGs. These enriched pathways may contribute to the metabolic regulation of PTB infection progression. Detailed protein expression analyses should be performed to verify the underlying mechanisms.

Using transcriptional profiling, we illustrated the metabolic profiles of the three patient groups. Surprisingly, glyoxylate and dicarboxylate metabolism, histidine metabolism, and pyruvate metabolism were enriched in the PTB versus HC differentially detected metabolites. Our results indicated that metabolism may be an important regulatory mechanism in PTB infection. When we compared the PTB_DM group with the PTB-only group, we found that some carbohydrate metabolism pathways were significantly enriched. Peripheral levels of some metabolites, such as acetone and phenol sulfate, may serve as monitoring factors for the disease. Some reports have also identified enriched pathways in PTB infections (27, 28). M. tuberculosis is thought to rely preferentially on fatty acid metabolism to establish and maintain chronic infections (28). In addition, altered glucose phosphorylation metabolism in DM is an important pathway in PTB infection (29, 30). The correlation between metabolite levels and disease progression should be confirmed in future studies.

Our combination analysis revealed that some shared DEGs and associated metabolites, such as *NOTCH1*, *STAT5A*, *HOMER3*, *SLC6A12*, *SP1*, *KCNE3*, and *FZD2*, were positively correlated with glutamic acid. Glutamic acid metabolism is thought to play an important role in PTB infection (31). NOTCH1 and STAT5 have also been reported to be involved in amino acid metabolism to prevent insulin resistance, which contributes to DM progression (32). *HPMER3* is associated with T cell regulation and was also enriched in our transcriptional enrichment analysis (33). Other genes are also associated with amino acid metabolism (34–37). Both our results and those of previous studies indicate that the NOTCH1/STAT pathway is an important regulatory pathway in PTB-infected patients with DM. However, how this pathway and its associated metabolites can be used as clinical biomarkers to monitor PTB progression requires further investigation.

The transcriptional profiles of patients with PTB were different from those of patients with PTB combined with DM. In addition, correlated metabolomic analysis revealed different metabolic profiles in peripheral whole-blood samples. Our combined analysis results showed that the NOTCH1/STAT pathway may be an important regulatory pathway in PTB-infected patients with DM and may be a potential therapeutic target for patients with PTB and DM.

## MATERIALS AND METHODS

### Clinical sample collection.

**FIG 10:**
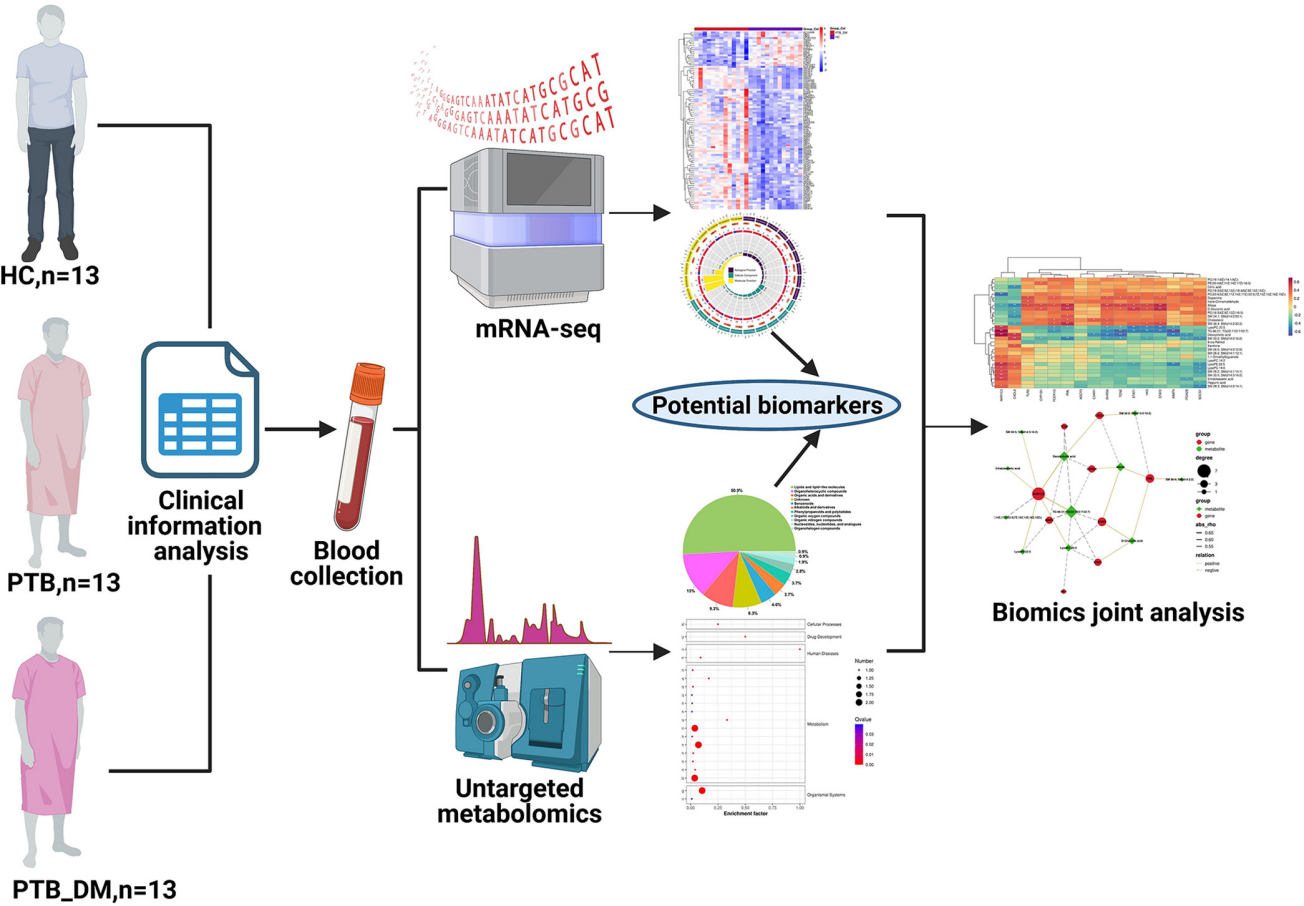
Experimental flowchart. Whole-blood samples from three groups of patients were used to perform mRNA sequencing and untargeted metabolomics. Subsequently, potential biomarkers were identified and biomics joint analysis was performed.

The collection of all blood samples and clinical information was approved by the ethics committee of Hangzhou Red Cross hospital (no. 2020-200). The subjects were divided into three groups: healthy controls (HC; 13 cases), patients with pulmonary tuberculosis (PTB; 13 cases), and patients with pulmonary tuberculosis and type 2 diabetes mellitus (PTB_DM; 13 cases). Based on the inclusion and exclusion criteria, we screened candidates and recruited subjects according to the following criteria: (i) standard for admission of healthy controls, healthy volunteers; (ii) standard for admission of patients with latent TB infection, infected patients with no symptoms associated with cough, sputum, fever, etc., no abnormalities on chest imaging, negative enzyme-linked immune spot test, positive interferon release test and tuberculin skin test, and no basis for TB activity; (iii) standards for admission of patients with TB (1); and (iv) standards for admission of patients with type 2 DM, (a) those with typical diabetic symptoms (polyuria, polyhydramnios, and unexplained weight loss), arbitrary blood glucose of ≥11.1 mmol/L, or fasting blood glucose ≥11.1 mmol/L and (b) age between 18 and 65 years. Subjects were excluded if they did not meet the inclusion criteria or if they met the following conditions: (i) urine protein quantification of >3.5 g/24 h; (ii) primary glomerulonephritis or secondary nephritis other than diabetic nephropathy; (iii) urinary protein positivity owing to urinary infection; (iv) coexisting serious primary diseases such as of the heart, brain, lung, liver (alanine aminotransferase [ALT] ≥1.5 times the upper limit of normal value), and hematopoietic system; (v) coexisting hepatitis, AIDS, rheumatism, or autoimmune diseases; (vi) coexisting malignant diseases; (vii) poorly controlled hypertension or secondary hypertension; (viii) history of diabetic ketoacidosis; (ix) severe infections within the previous 2 weeks; (x) allergy; (xi) pregnancy, lactation, or planning to become pregnant; (xii) participation in other clinical trials within 3 months; (xiii) disabilities classified by law (blind, deaf, mute, mentally handicapped, or physically handicapped); (xiv) suspected or confirmed history of alcohol or drug abuse or, according to the judgment of the investigator, a condition that predisposes the missing of visits (such as frequent changes in the work environment); and (xv) judgment of the investigator that the subject’s compliance would be poor. Whole-blood samples from the three subject groups were used for mRNA sequencing and untargeted metabolomics. Subsequently, potential biomarkers were identified, and a joint biomics analysis was performed. The experimental flowchart is shown in Fig. 10.

### RNA extraction and library construction.

Total RNA from each sample of peripheral blood was isolated and purified using TRIzol reagent (Invitrogen, Carlsbad, CA, USA) following the manufacturer’s instructions. The RNA amount and purity of each sample were quantified using a NanoDrop ND-1000 (NanoDrop, Wilmington, DE, USA). RNA integrity number was assessed using a Bioanalyzer 2100 (Agilent, CA, USA) with an RIN number of >7.0 and confirmed by electrophoresis on a denaturing agarose gel. Poly(A) RNA was purified from 1 μg of total RNA using Dynabeads oligo(dT) 25-61005 (Thermo Fisher, CA, USA) using two rounds of purification. Then the poly(A) RNA was fragmented into small pieces using a magnesium RNA fragmentation module (New England Biolabs [NEB], USA; catalog number e6150) at 94°C for 5 to 7 min. The cleaved RNA fragments were reverse transcribed into cDNA using SuperScript II reverse transcriptase (Invitrogen, USA; catalog number 1896649) and were then used to synthesize U-labeled second-stranded DNAs with Escherichia coli DNA polymerase I (NEB; catalog number m0209), RNase H (NEB; catalog number m0297), and dUTP solution (Thermo Fisher; catalog number R0133). An A-base was then added to the blunt ends of each strand to prepare them for ligation to indexed adapters. Each adapter contained a T-base overhang for ligation to the A-tailed fragmented DNA. Single- or dual-index adapters were ligated to the fragments and size selection was performed using AMPureXP beads. After treatment of the U-labeled second-stranded DNAs with heat-labile UDG enzyme (NEB; catalog number m0280), the ligated products were amplified by PCR using the following cycling conditions: initial denaturation at 95°C for 3 min; 8 cycles of denaturation at 98°C for 15 s, annealing at 60°C for 15 s, and extension at 72°C for 30 s; and then a final extension at 72°C for 5 min. The average insert size of the final cDNA library was 300 ± 50 bp. Finally, we performed 2 × 150-bp paired-end sequencing (PE150) on an Illumina NovaSeq 6000 (LC-Bio Technology Co., Ltd., Hangzhou, China), following the manufacturer’s recommended protocol. Fastq files for RNA-Seq are available from the NCBI SRA under project accession number PRJNA971365.

### Bioinformatics analysis of RNA-Seq.

Sequencing quality was also verified using Fastp (38), which was used to remove adapters, low-quality reads, and undetermined reads using default parameters. We used HISAT2 (39) to perform read mapping using the reference genome of Homo sapiens GRCh38. Mapped reads were assembled using StringTie (40) with the default parameters. All transcriptomes were then merged using Equation (41). StringTie was used to analyze mRNA expression levels by calculating fragments per kilobase of exons per million (FPKM) mapped fragments. The differentially expressed mRNAs were selected based on a fold change of >2 or <0.5 and with a parametric F test comparing nested linear models (*P* value < 0.05) using the R package edgeR (42).

### Metabolite extraction.

The collected serum samples were thawed on ice, and a 50% methanol buffer was used to extract the metabolites. The samples were stored at −80°C prior to LC-MS analysis. Pooled QC samples were prepared by combining 10 μL of each extraction mixture.

### UPLC and tandem mass spectrometry.

All chromatographic separations were performed using an ultra performance liquid chromatography (UPLC) system (SCIEX, UK). An ACQUITY UPLC T3 column (100 mm by 2.1 mm, 1.8 μm; Waters, UK) was used for reverse-phase separation. A TripleTOF 5600+ high-resolution tandem mass spectrometer (SCIEX, UK) was used to detect the metabolites eluted from the column. The quadrupole time of flight (Q-TOF) mass spectrometer was operated in both positive- and negative-ion modes. Furthermore, to evaluate the stability of the LC-MS during the entire acquisition, a quality control sample (pool of all samples) was acquired after every 10 samples.

### Metabolomics data analysis.

Pretreatments of the acquired MS data, including peak picking, peak grouping, retention time (RT) correction, second peak grouping, isotopes, and adduct annotation, were performed using XCMS software. Raw LC-MS data files were converted into mzXML format and then processed by the XCMS, CAMERA, and metaX packages using the R language (43–45). Each ion was identified by combining RT and *m/z* data. The intensity of each peak was recorded, and a three-dimensional matrix containing arbitrarily assigned peak indices (retention time-*m/z* pairs), sample names (observations), and ion intensity information (variables) was generated. Online KEGG database and Human Metabolome Database (HMDB) databases were used to annotate metabolites by matching the exact molecular mass data (*m/z*) of the samples with those from the databases, and an in-house fragment spectrum library of metabolites was used to validate the identification of metabolites. The intensities of the peaks were further preprocessed using metaX. Features that were detected in less than 50% of the QC samples or 80% of the biological samples were removed, and the remaining peaks with missing values were imputed using the k-nearest neighbor algorithm to further improve the data quality. PCA was performed for outlier detection and batch-effect evaluation using a preprocessed data set. Quality control-based robust locally estimated scatterplot smoothing (LOESS) signal correction was fitted to the QC data with respect to the order of injection to minimize signal intensity drift over time. In addition, the relative standard deviations of the metabolic features were calculated across all QC samples, and those of >30% were then removed. Student’s *t* tests were conducted to detect differences in metabolite concentrations between the two phenotypes, and the *P* value was adjusted for multiple tests using a false-discovery rate (FDR) (Benjamini-Hochberg). Supervised partial least-squares discriminant analysis (PLS-DA) was conducted using metaX to discriminate the different variables between the groups, and the altered metabolites between the two groups were identified by variable importance in the projection (VIP) of >1 from the PLS-DA and statistical analysis (*P* < 0.05). A total of 200 permutation tests were performed to investigate the quality of the model. The altered metabolites were analyzed using metabolomic pathway analysis (http://www.metaboanalyst.ca/) and correlated with potential pathways. The KEGG database was used to identify the functions of these metabolites in various metabolic pathways. Metabolomics raw data are uploaded to MetaboLights under project accession number MTBLS7623.

### Joint analysis of metabolites and transcripts.

Edge R was used for differential gene expression. The criteria for differentially expressed genes (DEGs) were |log_2_ fold change| of >1 and FDR of <0.05. The Cytoscape plugin “Metscape” was used to analyze the connected networks of differentially expressed metabolites and transcripts.

### Statistical analysis.

Two-tailed Student’s *t* tests were used to analyze the significance between two groups, and one-way analysis of variance (ANOVA) was used to compare more than two groups. Statistical significance was set at a *P* value of <0.05.

### Ethics approval.

This study was approved by the ethics committee of Hangzhou red cross hospital (approval no. 2020-200). All subjects recruited provided their written informed consent. The ethics committee of Hangzhou Red Cross Hospital has approved this consent procedure. In addition, the experiments in this study also conform with the Code of Ethics of the World Medical Association (Declaration of Helsinki).

### Data availability.

The data that support the findings of this study are available from the corresponding authors upon reasonable request. Fastq files for RNA-Seq are available from the NCBI SRA under project accession number PRJNA971365. Metabolomics raw data are uploaded to MetaboLights under project accession number MTBLS7623.
